# Antibacterial and Antibiofilm Properties of Postbiotics Derived from *Lactiplantibacillus pentosus* B1

**DOI:** 10.3390/ijms26178169

**Published:** 2025-08-22

**Authors:** Marta Nowak-Lange, Katarzyna Niedziałkowska, Aleksandra Tończyk, Carola Parolin, Beatrice Vitali, Katarzyna Lisowska

**Affiliations:** 1 Department of Industrial Microbiology and Biotechnology, Faculty of Biology and Environmental Protection, University of Lodz, 12/16 Banacha Street, 90-237 Lodz, Poland; katarzyna.niedzialkowska@biol.uni.lodz.pl (K.N.); aleksandra.tonczyk@biol.uni.lodz.pl (A.T.); 2 BioMedChem Doctoral School of University of Lodz and Lodz Institutes of Polish Academy of Sciences, 21/23 Matejki Street, 90-237 Lodz, Poland; 3 Department of Pharmacy and Biotechnology, University of Bologna, 40127 Bologna, Italy; carola.parolin@unibo.it (C.P.); b.vitali@unibo.it (B.V.)

**Keywords:** antimicrobial activity, biofilm, cell-free supernatant, lactic acid bacteria, organic acids

## Abstract

Lactic acid bacteria (LAB) are a promising source of postbiotics with potential applications in the cosmetic industry; however, strains isolated from fermented vegetables are rarely studied. This study aimed to characterize the bioactivity of postbiotics produced by *Lactiplantibacillus pentosus* B1 isolated from fermented beetroot juice. An organic extract (ELCFS) and a lyophilizate (LLCFS) were prepared from cell free supernatant of B1 and assessed for antimicrobial activity (MIC, MBC), biofilm inhibition and eradication ability against *Staphylococcus aureus*, *Escherichia coli*, *Streptococcus pyogenes* and *Cutibacterium acnes*. Stability (temperature, time) and cytotoxicity were also examined. Metabolite composition was determined by GC-MS. MIC values were 10 g/L for ELCFS and 10–20 g/L for LLCFS. ELCFS completely inhibited biofilm formation at 10 g/L, and LLCFS at 25 g/L; partial inhibition was observed at lower concentrations (e.g., *E. coli*—32.99% at 1 g/L ELCFS; *S. aureus* and *S. pyogenes*—74.01% and 95.67%, respectively, at 5 g/L). Complete eradication of mature biofilm was obtained at 2.5 g/L (ELCFS) and 12.5 g/L (LLCFS), while a significant partial effect was observed from 0.04 g/L ELCFS for *E. coli* (29.3%) and 0.2 g/L LLCFS for *S. pyogenes* (23.2%). *C. acnes* showed the lowest sensitivity. A reduction in viability of eucaryotic cells was observed at ≥ 5 g/L ELCFS (90.32%) and 12.5—50 g/L LLCFS (55.87—89.20%). Importantly, concentrations causing partial inhibition and eradication of biofilm did not show cytotoxicity towards fibroblasts. The preparations were thermostable and retained activity over time; only incubation of ELCFS at elevated temperature significantly reduced its antimicrobial activity against the *C. acnes* strain. GC-MS analysis revealed five organic acids, with lactic acid dominating. The results confirm the potential of *L. pentosus* B1 as a source of stable, effective, and safe postbiotics for cosmetology applications.

## 1. Introduction

The skin is the largest human organ, whose main function is to protect against microorganisms, toxins, and harmful environmental factors. In addition, it harbors a complex mixture of microorganisms that include bacteria, fungi and viruses, most of which are harmless or even beneficial to the host. The composition of the skin microbiota depends on gender, age, environment, skin characteristics (e.g., temperature, humidity, number of sebaceous glands), and host genotype [1,2]. Over the past decade, the taxonomic composition and diversity of the skin microbiome have been intensively studied in different populations. In adults with healthy skin, the microbiota shows relative stability and can contribute to maintaining homeostasis and modulating the inflammatory response, affecting almost all aspects of skin function. On the other hand, dysbiosis of the skin microbiota is often associated with the development of various dermatoses [3,4,5,6].

Microorganisms adapt to living on skin surfaces by inducing metabolic changes, including the production of exopolysaccharides, which facilitate biofilm formation. The biofilm is an organized community of microorganisms surrounded by an extracellular matrix, which provides them with enhanced survival and antibiotic resistance [2]. Studies on skin microorganisms, including species such as *Staphylococcus aureus*, *Cutibacterium acnes*, *Streptococcus pyogenes*, *Staphylococcus epidermidis* and *Malassezia* spp. have elucidated many of the mechanisms of their interaction with the host [2,7,8,9]. Numerous reports have shown an association between the presence of these microorganisms and inflammatory skin diseases such as acne vulgaris, atopic dermatitis (AD), psoriasis, rosacea, seborrheic dermatitis (SEA), boils and impetigo [10,11]. The etiology of these diseases is well understood and involves the initiation of biofilm on the epidermis or soft tissues, which is confirmed by numerous studies on the possibilities of its inhibition [12,13,14,15,16]. The growing risk of antibiotic resistance poses a significant public health challenge and leads to the search for new alternative therapeutic strategies. The use of antibiotics can result in an imbalance of the microbiota, promoting superinfection and other complications [17,18]. Therefore, dermatology and cosmetology are striving to develop therapies that selectively eliminate pathogens while maintaining skin microbial homeostasis.

With the increasing demand for natural and microbiota-friendly cosmetics, LAB have received considerable attention as potential active ingredients in formulations aimed at treating acne, AD, photo-aging or skin sensitivity [19]. Probiotics, which include LAB, have been defined by the Food and Agriculture Organization of the United Nations and the World Health Organization as ”iving microorganisms that, when provided in adequate amounts, result in health benefits for the host” [20]. Numerous clinical studies have illustrated that the consumption of probiotics has positive effects on the gut, nervous system, metabolism, immunity, etc. [21,22,23]. Due to these beneficial effects, interest in probiotics in various industries has increased. In particular, the use of probiotics in skin care products has emerged as a trend with beneficial effects on the skin microbiota. Natural products are preferred by the majority of the consumers for the treatment of various skin diseases compared to conventional therapy [24,25]. Although probiotics have numerous benefits, safety issues. Sepsis, endocarditis or bacteremia in immunocompromised individuals and also the acquisition and transmission of antibiotic resistance genes have received attention in recent years [26].

Consequently, the idea has emerged that microbial viability is not essential for probiotics to have a beneficial effect on human health [27]. The term “postbiotics” has therefore been proposed, the precise definition of which has been developed by the International Scientific Association of Probiotics and Prebiotics as “a preparation of non-living microorganisms and/or their components that provides health benefits to the host” [28]. Postbiotics, including microbial metabolites, short-chain fatty acids, antimicrobial peptides, cell lysates and other components, offer several technological advantages: stability, safety, ease of storage and biological activity [29,30]. The use of postbiotics in dermatology is supported by a growing body of evidence from preclinical and clinical studies that have demonstrated their potential in the treatment of a range of skin conditions, from acne to eczema. For example, Chung et al. (2022) [19] developed a multi-strain postbiotic complex combining cell free supernatants (CFS) of *Lactobacillus helveticus* HY7801 and *Lactococcus lactis* HY449. This complex demonstrated antimicrobial activity against *S. aureus* and *C. acnes*, and modulated the levels of inflammatory cytokines and hyaluronic acid, supporting an anti-inflammatory response in skin cells. *Lactobacillus reuteri* lysate has also been shown to reduce skin inflammation and promote wound healing (Han et al., 2019). Rice flour fermented with *Lactobacillus paracasei* CBA L74 was found to be effective in the treatment of atopic dermatitis in infants [31]. Postbiotics from *Lactobacillus* sp. RM1 showed antifungal activity against *Aspergillus parasiticus* and *Aspergillus carbonarius*. 2-methyldodecane and pentadecane have been identified as antifungal compounds in postbiotics [32].

Pickled beetroot juice, also known as beetroot sourdough, a popular food product in Poland, is a natural source of LAB and their metabolites. Its spontaneous fermentation results in the formation of complex consortia of microorganisms, including LAB, which makes it an interesting material for the search of new bioactive compounds [33,34].

The aim of the present study was to characterize postbiotics produced by a strain of *Lactiplantibacillus pentosus* (B1), isolated from beetroot sourdough, and to evaluate their antimicrobial and antibiofilm potential, excluding cytotoxic reactions. The postbiotic preparations, obtained by organic extraction and lyophilization, were subjected to qualitative and quantitative analysis (GC-MS), and their stability was assessed by thermal assays. This is the first known study to use a *Lactiplantibacillus pentosus* B1 strain, isolated from beetroot sourdough, to produce postbiotics with documented antimicrobial and antibiofilm activity. In addition, a comparison was made between the effects of two different postbiotic preparation methods on their biological activity.

## 2. Results and Discussion

### 2.1. Isolation and Identification of LAB Isolates

Pickled beetroot juice obtained from a local farm, which was free from preservatives, synthetic chemicals and pesticides, was used to isolate LAB. After inoculating the samples onto de Man, Rogosa and Sharpe (MRS) agar and incubating them under microaerophilic conditions, numerous colonies of varying morphology were obtained. One of these isolates was selected for further analysis and designated strain B1. This isolate was chosen based on its superior viability under cultivation conditions, as well as preliminary screening studies that indicated antimicrobial activity against reference bacterial strains. The procedure of molecular identification, performed using the BLAST (http://blast.ncbi.nlm.nih.gov, Access: 02 February 2024) tool and the EzBioCloud platform, described in the Materials and Methods Section, revealed a 100% sequence identity with *Lactiplantibacillus pentosus* 124-2 (DSM 20314, ATCC 8041) based on 16S rRNA gene sequencing.

The dominance of LAB species, such as *Lactiplantibacillus*, *Leuconostoc*, and *Weissella*, in the spontaneous fermentation of plant substrates is well documented. LAB have been isolated from fermented African leafy vegetables [35], kimchi [36], and sauerkraut [37]. Importantly, *L. pentosus* has been recognized as a GRAS organism with known probiotic properties, including immunomodulation and antimicrobial activity [38].

The isolation of LAB from fermented beetroot juice stems from the growing interest in exploring unconventional plant sources for the isolation of functional strains. As demonstrated by Garcia et al. [39], LAB strains isolated from the pulp of fruits such as mango, pineapple, and wild strawberry can exhibit variable antimicrobial profiles depending on the species and study conditions. For example, *Lactiplantibacillus pentosus* strains exhibited measurable inhibition zones for foodborne and clinical pathogens, including *S. aureus*, *E. coli*, and *Listeria monocytogenes*. These observations are consistent with our preliminary results with strain B1.

The bioactive potential of this species, including the production of bacteriocins and organic acids, may explain the observed antimicrobial effects in preliminary tests and warrants further research into its potential as a candidate for applications in various industries, including the cosmetics industry.

### 2.2. Antimicrobial Activity of L. pentosus Postbiotics

For a long time, antibiotic therapy was the primary therapeutic approach for the treatment of skin diseases associated with microbial colonization. However, this approach carries a significant risk of disrupting the homeostasis of the skin microbiota, as it may lead to the elimination of not only pathogens but also commensal microorganisms [40]. In addition, the overuse of antibiotics, both in humans and animals, has led to a high prevalence of antimicrobial-resistant microorganisms that can become ”uperbacteria”. Pickled vegetables are rich in beneficial LAB due to the high nutrient content of these foods. Many studies have assessed the health benefits associated with these microorganisms, including the inhibition of pathogen growth and biofilm formation by producing antimicrobial substances or competing with pathogenic bacteria for nutrients [21,31,39,41]. Due to the potential risks associated with the application of live microorganisms, especially in immunocompromised individuals, growing research interest has turned towards postbiotics—non-living products of bacterial metabolism that retain benefical biological effects without the risk of complications [42].

The aim of this study was to evaluate the biological properties of postbiotics obtained from the *Lactiplantibacillus pentosus* B1 strain isolated from beetroot sourdough. After 24 h of culture in MRS broth, the pH of the post-culture fluid was measured (3.58), and the organic extract (ELCFS) and lyophilisate (LLCFS) were prepared from it.

**Figure 1:**
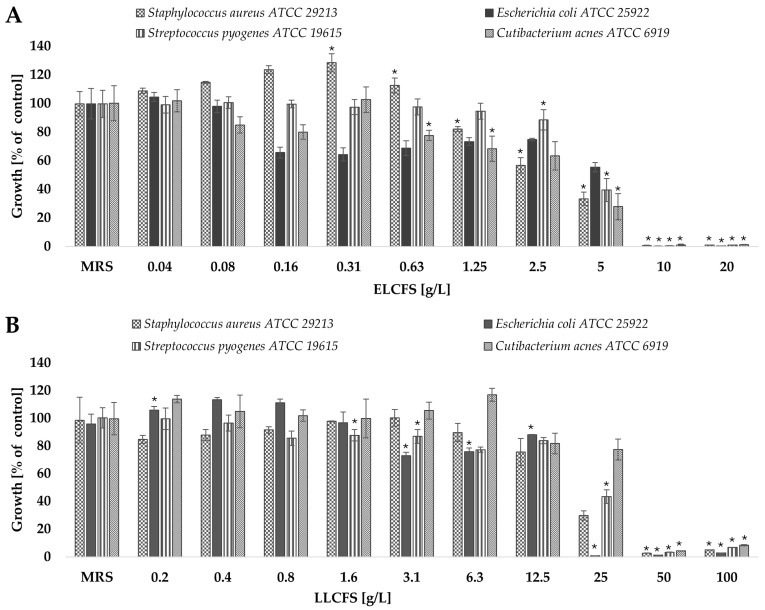
Antimicrobial activity of the obtained postbiotics against four bacterial strains: (**A**) ELCFS, (**B**) LLCFS. The results are presented as mean bacterial growth values (a percentage of control) ± standard deviation (SD) (*n* = 4). Statistically significant differences are indicated as * (*p* < 0.05).

Antimicrobial activity was assessed by microdilution in Muller-Hinton (MH) broth and Brain Heart Infusion (BHI) broth against Gram-positive (*S. aureus* ATCC 29213, *S. pyogenes* ATCC 19615, *C. acnes* ATCC 6919) and Gram-negative (*E. coli* ATCC 25922) strains. The concentration range tested was 0.04–20 g/L for the ELCFS and 0.2–100 g/L for the LLCFS (Figure 1).

The results showed that the postbiotics demonstrated antimicrobial activity against all the bacterial strains tested, and that this effect depended on the concentration used. To ensure the specificity of the observed effects, control samples of culture media that had undergone identical extraction and lyophilisation procedures, were included in all tests. These controls showed no antimicrobial activity, confirming that the observed effects were due to metabolites produced by LAB.

ELCFS exhibited significantly stronger antimicrobial activity than LLCFS, inhibiting microbial growth at lower concentrations. *E. coli* was found to be the most sensitive to ELCFS, with growth reduced by 34.36% at a concentration of 0.16 g/L. A concentration of 5 g/L resulted in statistically significant inhibition (*p* < 0.05, *n* = 4) of the growth of all tested microorganisms (*S. aureus*, *E. coli*, *S. pyogenes* and *C. acnes*) by 66.67%, 44.51%, 60.55% and 72.20%, respectively. Concentrations of ≥10 g/L resulted in the complete inhibition of growth for all tested strains.

The antimicrobial effect of LLCFS was noticeably weaker. Only at a concentration of 25 g/L was a significant reduction in microbial growth observed: 70.20% for *S. aureus*, 99.17% for *E. coli*, 56.70% for *S. pyogenes* and 22.57% for *C. acnes* (*p* < 0.05, *n* = 4). Complete growth inhibition was observed at concentrations of ≥50 g/L.Consistency with our observations is confirmed by numerous publications on postbiotics of LAB origin. For example, the active components of CFS of *Lactobacillus pentosus* L-36 strain showed significant antimicrobial activity against clinical and reference strains of *S. aureus*, inducing growth inhibition zones with diameters ranging from 16.67 to 22.38 mm in the disk diffusion test [43]. Almeida et al. (2024) [44] also confirmed the efficacy of supernatants from *Lactiplantibacillus plantarum* strains A2 and 2.1 against *E. coli* ATCC 25922, using both the diffusion test and the microplate method. In an analysis using 96-well microtitre plates, LPA2 showed 89.22% inhibition of bacterial growth, while LP2.1 showed 91.22%. Rather et al. (2022) [45] described the efficacy of *L. plantarum* KAU007 metabolite extract against *S. pyogenes* ATCC 8668 at low doses. Chae et al. (2021) [46] investigated the effect of different concentrations of CFS from *L. plantarum* strains APsulloc 331261 and APsulloc 331266, isolated from green tea, on the growth of *S. aureus* ATCC 6538, *C. acnes* ATCC 6919, and the fungi *Candida albicans* ATCC 90028, *Malassezia globosa* MYA-4612, and *Malassezia restricta* MYA—4611. The data obtained confirmed significant antimicrobial activity, which was dose-dependent. Supernatants at a concentration of 50% inhibited the growth of *S. aureus* by 97.7% (APsulloc 331261) and 98.6% (APsulloc 331266), while concentrations in the range of 2.5—25% resulted in a reduction in growth of 21–31% and 21—29%, respectively. In the case of *C. acnes*, concentrations of 25% and 50% led to growth inhibition of 74.4% and 96.5% (APsulloc 331261) and 98.8% and 99.9% (APsulloc 331266), respectively. For *C. albicans*, concentrations of 75—100% resulted in growth reduction in the range of 37.3—65.4% (APsulloc 331261) and 61.9—75.8% (APsulloc 331266). Complete inhibition of the growth of *M. globosa* and *M. restricta* was achieved with 100% supernatant from both tested strains.

In the present study, the values of the minimum inhibitory concentration (MIC) and the minimum bactericidal concentration (MBC) were also determined. Postbiotics in the form of ELCFS showed a higher antimicrobial activity, with a consistent MIC value of 10 g/L for all strains tested. In contrast, the MIC for LLCFS was 25 g/L against *E. coli*, and 50 g/L for the other microorganisms. The MBC for ELCFS was the lowest against *E. coli* (10 g/L), while for the other strains its value was 20 g/L. For LLCFS, *E. coli* also proved to be the most sensitive strain (MBC = 50 g/L), while for *S. aureus* and *C. acnes* the MBC was 100 g/L. For *S. pyogenes*, the MBC exceeded the upper range of the tested concentrations (>100 g/L). Importantly, MIC and MBC values determined after 12 months of storage of both ELCFS and LLCFS at –20 °C remained unchanged, confirming the long-term stability of the antimicrobial activity of the tested postbiotics (Table 1).

Postbiotics have shown a significant antimicrobial activity against a broad spectrum of pathogens, suggesting their potential use in the food, pharmaceutical and cosmetic industries. In comparison, Wang & Zeng (2022) [43] revealed that *Lactobacillus pentosus* L-36 culture supernatants possessed antimicrobial activity against *S. aureus*, responsible for mastitis in cattle, with an MIC value of 31.25 g/L. Xu et al. (2024) [47] described the efficacy of *Lacticaseibacillus paracasei* LPH01 postbiotics in eliminating bacteria involved in the pathogenesis of acne, setting a MIC value of 12.5 g/L against *C. acnes*. In addition, Jin et al. (2025) [48] isolated the strain *Pediococcus pentosaceus* from ginseng, whose supernatant showed potent activity than our postbiotics, against skin bacteria: a MIC in the range of 1.25–2.5 g/L for *S. epidermidis*, 0.625 g/L for *S. aureus*, and MBC of 5 g/L for both strains. Rather et al. (2022) [45] described the efficacy of the metabolite extract (LME) of *L. plantarum* KAU007 against *Streptococcus pyogenes* ATCC 8668. The studies showed high efficacy of LME at very low concentrations, with a MIC of 9.76 μg/mL and a MBC of 39.06 μg/mL. In relation to these data, the activity of *L. pentosus* B1 postbiotics against *C. acnes* and *S. pyogenes* remains at a competitive level, especially in the form of ELCFS. Although in vitro activity does not always translate directly into in vivo efficacy, the results obtained confirm the promising therapeutic potential of *L. pentosus* B1 postbiotics in the context of skin infections. However, further studies—including skin model tests, analysis of mechanisms of action and evaluation of safety of use—are needed to confirm this hypothesis.

The results clearly indicate that postbiotics obtained by organic extraction show a higher antimicrobial activity than their lyophilised counterparts. Literature data confirm that the biological activity of postbiotics can vary significantly depending on the method used to obtain them. The use of ethyl acetate extraction makes it possible to obtain more concentrated preparations containing, among other things, organic acids and other metabolites. This may explain the stronger antimicrobial activity of ELCFS compared to LLCFS [49].

The effect of elevated temperature on the stability of the postbiotics was assessed in this study by determining the percentage residual activity (%RA) according to the method described in the Methods Section. The results obtained, presented in Table 2, showed that incubation of ELCFS at elevated temperature significantly reduced its antimicrobial activity only against the *C. acnes* strain. The residual activity of ELCFS against this strain was 29.66%, 30.77% and 50.29% after a 30 min incubation at 40, 60 and 80 °C, respectively, compared to the control stored at refrigeration temperature (4 °C).

LLCFS showed only a slight, but statistically significant, reduction in the antimicrobial activity against *C. acnes*—the residual activity after incubation at 40, 60 and 80 °C reached 97.8%, 95.8% and 96.4%, respectively. The data obtained are consistent with previous reports. Almeida et al. (2024) [44] confirmed the thermostability of *L. plantarum* A2 and *L. plantarum* 2.1 postbiotics active against *E. coli*. Heating at 100 °C for 15 min, as well as treatment with trypsin (2.5 mg/mL), did not abolish their inhibitory activity. Similar results were obtained by Singh et al. (2019) [2], who evaluated the stability of postbiotics derived from cultures of *Bacillus amyloliquefaciens* J and *Lactobacillus plantarum* SN4—their antimicrobial activity was fully preserved even after exposure to 121 °C. The stability of postbiotics under refrigeration and room conditions is a key parameter from the point of view of their potential commercial use in pharmaceutical and cosmetic formulations.

### 2.3. Antibiofilm Activity of Postbiotics of L. pentosus

The biofilm-forming ability of *S. aureus*, *E. coli*, *S. pyogenes* and *C. acnes* strains was investigated in the presence of postbiotics in the form of ELCFS and LLCFS. Two mechanisms of action of the postbiotics were assessed: inhibition of biofilm formation and eradication of biofilm already formed. For the biofilm formation inhibition test, concentrations corresponding to MIC values and 0.9 × MIC, 0.8 × MIC, 0.5 × MIC, 0.1 × MIC and 0.01 × MIC were used. For the eradication test, the same ranges of concentrations were used as for the antimicrobial activity test: 0.04–20 g/L for ELCFS and 0.2–100 g/L for LLCFS.

#### 2.3.1. Biofilm Inhibition

**Figure 2:**
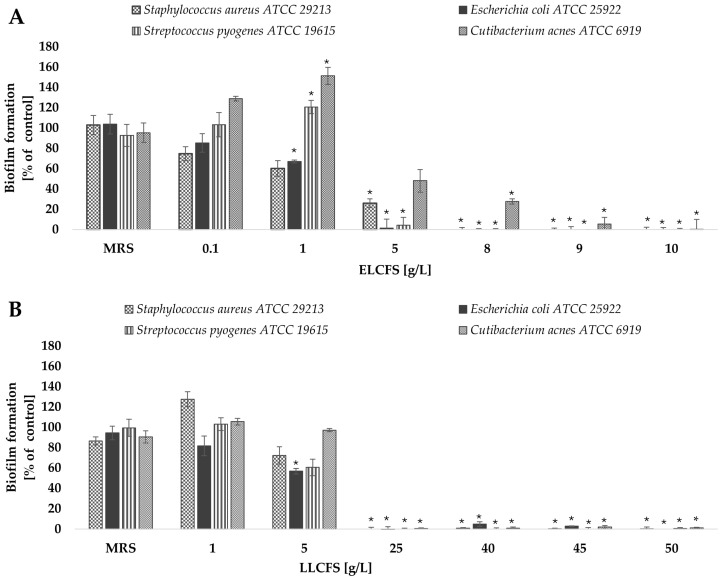
Effect of ELCFS (**A**) and LLCFS (**B**) on biofilm formation by the bacterial strains tested. The results are presented as a percentage of control ± SD (*n* = 4). Statistically significant differences were denoted as * (*p* < 0.05).

The first aspect analyzed was the ability of ELCFS and LLCFS to limit the formation of biofilm structures in the initial phase of their development. The results are presented as the percentage intensity of biofilm formation relative to the biotic control (not treated with postbiotics) (Figure 2).

Analysis of the effect of ELCFS and LLCFS on biofilm formation showed a clear concentration-dependence for all tested strains. In the case of ELCFS, the highest sensitivity was observed for *E. coli*, where a significant inhibition of biofilm formation of 32.99 ± 1.37% (*p* < 0.05, *n* = 4) was achieved at a concentration of 1 g/L. For *S. aureus*, a significant inhibitory effect (74.01 ± 4.23%) was observed at a concentration of 5 g/L (*p* < 0.05, *n* = 4), while for the remaining strains full inhibition was achieved at 5–10 g/L.

For LLCFS, a significant reduction in biofilm formation in all tested strains was observed only at concentrations ≥ 25 g/L, achieving inhibition levels ranging from 99.40% to 100% (*p* < 0.05, *n* = 4). In most cases, the effective concentrations were higher than for ELCFS, confirming the greater effectiveness of the ELCFS fraction under the conditions tested. The concentrations of postbiotics required to inhibit biofilm formation were lower than the corresponding MIC values determined for planktonic cells. This suggests that the tested postbiotics may interfere with early stages of biofilm development, such as bacterial adhesion and quorum sensing, even at sub-inhibitory concentrations.

#### 2.3.2. Biofilm Eradication

**Figure 3:**
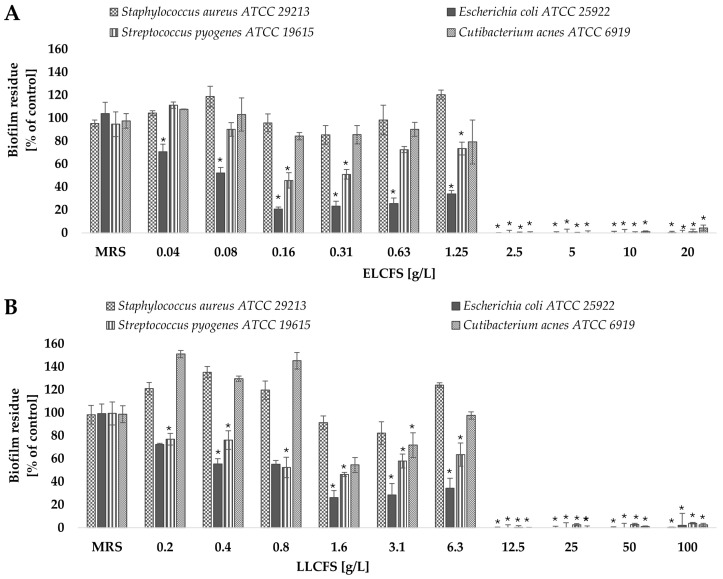
Effect of ELCFS (**A**) and LLCFS (**B**) on biofilm eradication of the tested bacterial strains. The results are presented as percentage of control ± SD (*n* = 4). Statistically significant differences were denoted as * (*p* < 0.05).

After assessing the ability of postbiotics to inhibit the early stages of biofilm formation, the next stage of the research was to analyze their effectiveness in eradicating already mature biofilm structures. Both the ELCFS and the LLCFS of *L. pentosus* postbiotics showed a strong eradication activity against the mature biofilm of all bacterial strains tested, in the concentration ranges of 2.5–20 g/L for ELCFS and 12.5–100 g/L for LLCFS (Figure 3). The greatest sensitivity to both preparations was observed for *E. coli* and *S. pyogenes* strains. In the case of *E. coli*, even the lowest concentration of ELCFS used (0.04 g/L) induced a biofilm dispersion of 29.3 ± 6.6% (*p* < 0.05, *n* = 4). LLCFS induced a statistically significant biofilm eradication of the *S. pyogenes* strain (23.2 ± 5.0%, *p* < 0.05, *n* = 4) even at the lowest concentration tested (0.2 g/L).

The *C. acnes* strain showed the lowest sensitivity among the tested bacteria—a significant eradication effect was observed only at concentrations ≥ 2.5 g/L for ELCFS and 12.5 g/L for LLCFS (*p* < 0.05, *n* = 4). The observed differences in eradication efficacy may be related to the architecture of the biofilm and the different sensitivity of the extracellular matrix components to postbiotics. The high efficacy against *E. coli* may be due to the less complex biofilm structure of this bacterium in vitro, compared to *S. aureus* or *C. acnes* biofilms.

#### 2.3.3. Confocal Microscopy

**Figure 4:**
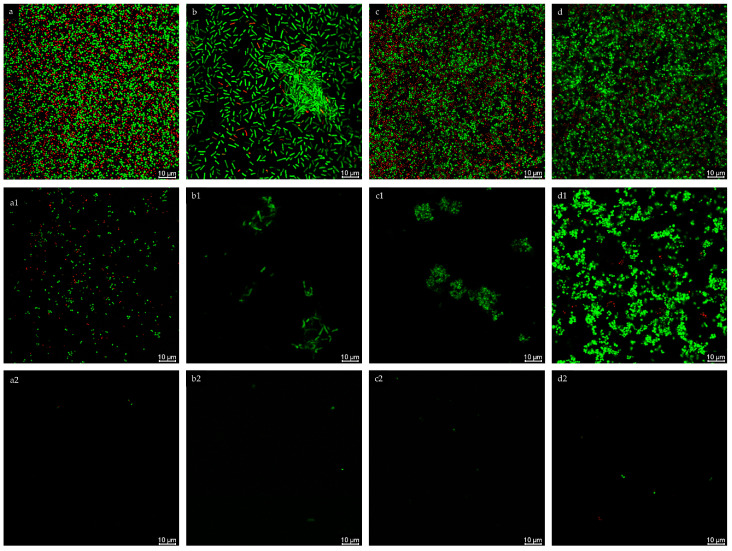
Confocal microscopy analysis of biofilm inhibition of *S. aureus* (**a**), *E. coli* (**b**), *S. pyogenes* (**c**), *C. acnes* (**d**) treated with ELCFS (1) and LLCFS (2). 10 µm scale. Confocal microscopy analysis of biofilm inhibition of *S. aureus* ((**a**): control, (**a1**): treated with ELCFS, (**a2**): treated with LLCFS); *E. coli* ((**b**): control, (**b1**): treated with ELCFS, (**b2**): treated with LLCFS); *S. pyogenes* ((**c**): control, (**c1**): treated with ELCFS, (**c2**): treated with LLCFS); *C. acnes* ((**d**): control, (**d1**): treated with ELCFS, (**d2**): treated with LLCFS). 10 µm scale.

The effect of postbiotics on the biofilm structure of the tested strains was also assessed using confocal laser microscopy and fluorescence staining to distinguish between live and dead cells. The analysis was performed using the postbiotic concentrations of 2.5 g/L for ELCFS and 25 g/L for LLCFS. Representative images of the biofilms are shown in Figure 4.

In the control samples, the biofilms of all strains exhibited a compact, three-dimensional structure, with numerous, densely arranged living cells and single dead cells. Following ELCFS treatment, a disruption of biofilm continuity and a reduction in cell number were observed. However, *E. coli* and *S. pyogenes* retained small, compact aggregates, while *C. acnes* formed extensive colonies, suggesting limited penetration of the extract into the biofilm matrix.

LLCFS caused significantly greater disruption of the biofilm structure—primarily single, scattered cells, both living and dead, were visible, with a marked reduction in the layer’s thickness and cohesion. This effect was consistent across all strains tested. Confocal observations confirmed the results of the quantitative tests, indicating greater efficacy of LLCFS in disrupting mature biofilms, while individual species exhibited varying susce tibility to ELCFS.

Skin diseases, including atopic dermatitis, various forms of chronic ulcers, acne, ankylosing spondylitis, necrotizing fasciitis, sweating disorders, cellulitis, erythema nodosum and rosacea, are usually caused by polymicrobial infections. In recent years, it has been confirmed that their etiology involves the initiation of bacterial accumulation and biofilm formation on the epidermal surface or within the soft tissues. Biofilm poses a serious public health threat due to the high resistance of pathogens to most standard antibiotics [2].

Our results confirm the antimicrobial potential of *L. pentosus* B1 postbiotics against skin pathogens, at both the formation and eradication stages of biofilm. The observed effect was concentration- and composition-dependent; the ELCFS were more effective at lower concentrations, while LLCFS were effective over a wider range of concentrations. Similar relationships were observed in the studies by other authors. For example, Yang et al. (2021) [50] evaluated the antimicrobial and antibiofilm activity of a CFS of *Lactobacillus reuteri* AN417 against the following oral pathogens: *Porphyromonas gingivalis* and *Streptococcus mutans*. They demonstrated that postbiotics significantly reduced biofilm formation at an early stage of bacterial colonization, a finding that was confirmed by fluorescence microscopy. Furthermore, treatment of 5-day-old biofilms with the *L. reuteri* CFS at the concentrations of 10%, 20%, and 30% resulted in their significant eradication and decreased the expression of genes involved in biofilm formation. Shangguan et al. (2021) [51] investigated the effect of *Lactobacillus paracasei* L10 CFS on *Vibrio parahaemolyticus* biofilms. They observed a concentration-dependent inhibition of biofilm formation (up to 57%) and a decrease in its metabolic activity (up to 47%) following the addition of 6% of the supernatant. Treating mature, 24 h-old biofilms with 24% supernatant for 4 h resulted in a 41% reduction in biomass and metabolic activity. Similarly to the present study, they demonstrated stimulation of biofilm formation in the presence of the lowest concentrations of the supernatant (below 3%). Fluorescence microscopy confirmed the decrease in metabolic activity and the presence of dead cells, while confocal microscopy revealed the destabilization of the formed biofilm structures. Finally, Huang et al. (2025) [52] described the efficacy of a mixture of CFS of different LAB. They found that the mixture was more effective at preventing biofilm formation than eradicating it. For *Pseudomonas aeruginosa*, the protective effect was stronger than the destructive effect, while *Listeria monocytogenes* required a high concentration (62.5 g/L) to inhibit biofilm formation. The obtained results taken together with literature reports indicate the potential of *L. pentosus* B1 postbiotics as promising candidates for use in antimicrobial strategies targeting skin pathogen biofilms.

### 2.4. Cytotoxic Activity of Postbiotics

**Figure 5:**
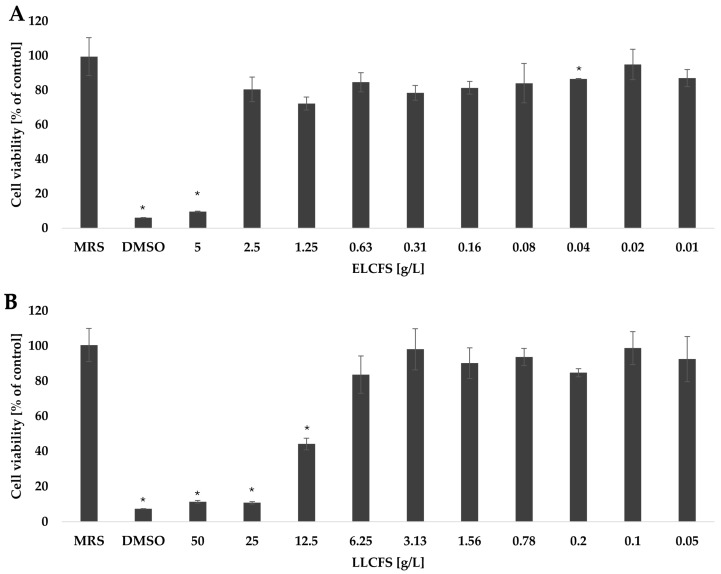
Survival of fibroblasts treated with ELCFS (**A**) and LLCFS (**B**). The results are presented as mean cell viability values (percentage of control) ± SD (*n* = 4). Statistically significant differences are indicated as * (*p* < 0.05).

High safety and stability of postbiotics are key prerequisites for their potential use in the cosmetics industry. The cytotoxic activity of the obtained postbiotics was assessed in vitro using mouse fibroblasts of the NCTC clone 929 line, in the concentration range of 0.01–10 g/L for ELCFS and 0.05–50 g/L for LLCFS (Figure 5). To verify that the observed effects were not due to the processing method or media components, appropriate control samples were included. In the case of the ELCFS, no significant effect on cell viability was observed in the concentration range from 0.01 to 2.5 g/L. The use of a concentration of 5 g/L resulted in a significant decrease in fibroblast viability by 90.32 ± 0.25% compared to the control (*p* < 0.05, *n* = 4), which indicates a clear cytotoxic effect at this dose.

The LLCFS showed better cellular tolerance. No significant reduction in fibroblast viability was observed at concentrations up to 6.25 g/L. A significant reduction in cell viability was noted only at higher concentrations: 12.5 g/L (88.70 ± 0.72%), 25 g/L (89.20 ± 0.61%) and 50 g/L (55.87 ± 3.32%) (*p* < 0.05, *n* = 4). According to the ISO 10993-5 guidelines [53], cell viability >80% indicates no cytotoxicity; values in the range of 80–60%, 60–40% and below 40% indicate weak, moderate and strong cytotoxicity, respectively. The dependence of the cytotoxic effect on the concentration was also confirmed for postbiotics from other LAB strains in the studies on different cell lines. Kim et al. (2024) [54] showed that the CFS of LK1 (Lentilactobacillus kefiri) at a concentration of 50% showed weak toxicity (62.71%) against Caco-2 colon adenocarcinoma cells, while the concentrations of 25%, 12.5% and 6.25% showed no cytotoxic effect. Similar observations were made with the lyophilized CFS of Lactobacillus casei, which did not negatively affect the viability of NIH/3T3 fibroblast cells at the concentrations of 6.25, 3.12 and 1.56 mg/mL [55]. However, due to the strain-dependent production of bioactive compounds by LAB, the cytotoxic effect may vary depending on the cell type. Jin et al. (2025) [48] found that the postbiotic Pediococcus pentosaceus THG-219 showed cytotoxic activity against human HaCaT keratinocytes only at the highest tested concentration (500 µg/mL), whereas no significant toxic effects were observed at concentrations up to 100 µg/mL.

### 2.5. Production of Postbiotics and Identification of Organic Acids

LAB can synthesize various classes of compounds with antimicrobial activity, including organic acids, fatty acids, bacteriocins, cyclic dipeptides, and phenolic compounds [56]. The thermostable nature of the postbiotics obtained in this study indicates that their activity may be primarily due to the presence of organic acids, which is consistent with the findings of Crowley et al. (2013) [57], who highlighted the heat resistance of organic acids produced by *Lactobacillus* strains. To identify the compounds responsible for antimicrobial activity, chemical analysis of ELCFS and LLCFS was performed using full-scan GC-MS. Based on retention times and mass spectra compared with the NIST 08 MS library, five major acids were identified: lactic, hydroxyisocaproic, succinic, shikimic, and malonic. Eight-point calibration curves for these compounds demonstrated good linearity (r = 0.997) in the range of 2–100 µg/mL. Quantitative analysis revealed clear differences in the content of individual acids between ELCFS and LLCFS. In all cases, except for shikimic acid, concentrations were higher in ELCFS. The highest concentration was found for lactic acid (ELCFS: 350.64 mg/L; LLCFS: 98.53 mg/L), while the remaining acids ranged from 1.56 to 12.63 mg/L (ELCFS) and 0.66–3.10 mg/L (LLCFS) (Table 3). These differences may be related to different sample preparation procedures. Solvent extraction (ELCFS) favors the concentration and recovery of compounds soluble in a given solvent, while freeze-drying (LLCFS) may result in partial loss of volatile metabolites or those sensitive to changes in temperature, pressure, or freezing/dehydration.

Although the concentrations of individual organic acids were lower than the MIC values determined for planktonic cells, the obtained results of antimicrobial and antibiofilm activity suggest synergistic effects between several metabolites. This phenomenon is well documented and may explain the greater effectiveness of postbiotic mixtures compared to individual compounds [58,59,60]. Synergy may result from the simultaneous activation of several mechanisms: lowering environmental pH, destabilizing the cell membrane, disrupting proton transport, limiting bacterial adhesion and modulating quorum-sensing systems. These results are consistent with the observations of Hu et al. (2019) [61] who identified five organic acids (lactic, acetic, tartaric, citric, malic) in CFS of *L. plantarum* strains and linked their presence with the activity against *S. aureus*. Similarly, Saidi et al. (2023) [62] showed that inactivation of bacteriocins did not significantly affect the antimicrobial activity against *S. aureus*, indicating the key role of organic acids. Jalali et al. (2024) [41] and da Costa et al. (2018) [56] confirmed that organic acids are the main factor in the antimicrobial activity of CFS LAB. In turn, Li ShuHong et al. (2019) [63] described their mechanism of action as a combination of increasing the permeability of cell membranes and acidification of the environment, which leads to bacterial cell death. In the case of lactic acid, increased adsorption of antimicrobial molecules and disruption of proton transport are also observed, conferring a strong antibacterial and antibiofilm effect [64]. Available literature data on the antimicrobial activity of individual organic acids indicate that these compounds are effective inhibitors of the growth and biofilm formation of various microorganisms. Chotigarpa et al. (2018) [65] demonstrated that the MIC and MBC values of lactic acid were 0.5% against *S. aureus* and *Staphylococcus epidermidis*. Amrutha et al. (2017) [66] determined the MIC values of acetic, citric, and lactic acids against *E. coli* (1.5%, 2% and 0.2%, respectively) and *Salmonella* spp. (1%, 1.5% and 1%, respectively). These studies also noted a significant reduction in biofilm formation, with the maximum inhibition of biofilm formation by *E. coli* being 39.13% when 2% lactic acid was used. Hydroxyisocaproic acid showed bactericidal activity against various species, including *C. acnes*, at a concentration of >160 g/L after 72 h [67]. Succinic acid, identified as the main active component of pickled and dried mustard extract, showed antimicrobial activity against *S. aureus* and *Pseudomonas fluorescens*, forming growth inhibition zones of 24.84 mm and 18.66 mm, respectively, at a concentration of 30 g/L extract [68]. Shikimic acid effectively inhibited *S. aureus* biofilm formation at concentrations below the MIC, reducing both biofilm biomass and the metabolic activity of biofilm cells [69]. Malonic acid showed MIC values of 0.5–0.75 mg/mL and MBC of 1.5 mg/mL against *Helicobacter pylori* ATCC 43504 and HPM001 [58].

The collected data support the hypothesis that the postbiotic activity of *L. pentosus* B1 results from the presence of a multicomponent profile of organic acids, the effects of which may be enhanced by synergistic effects.

Although this study focused on the analysis of organic acids, the significant contribution of other metabolites, such as bacteriocins, biosurfactants, and hydrogen peroxide, cannot be ruled out. It is worth noting that some *L. pentosus* strains simultaneously synthesize organic acids and broad-spectrum bacteriocins, such as plantaricins, which may interact with the acid fraction, enhancing the antimicrobial effect [70].

## 3. Materials and Methods

### 3.1. LAB Isolation and Identification

LAB strains were isolated from fermented beetroot juice from a regional farm. The diluted sample was plated on MRS agar medium (Becton Dickinson, Warsaw, Poland). The plates were incubated at 37 °C under microaerophilic conditions (6% O_2_, 10% CO_2_, 84% N_2_), obtained using the Anoxomat Mark II system (Mart Microbiology, BV, the Netherlands). Colonies with diverse morphology were isolated and deposited in theCollection of the Department of Industrial Microbiology and Biotechnology, University of Lodz. The strains were stored at −80 °C in MRS broth with 50% glycerol (*v*/*v* 1:1) (Chempur, Piekary Śląskie, Poland). One strain, designated as B1, was selected for further analysis. Its selection was based on high survival and growth potential in cultivation conditions, as well as promising antimicrobial activity observed in prior preliminary screening assays. Molecular identification was performed at the Laboratory of DNA Sequencing and Oligonucleotide Synthesis of the Institute of Biochemistry and Biophysics of the Polish Academy of Sciences (IBB PAN, Warsaw, Poland) based on sequencing of the 16S rRNA gene. Universal primers 27F 5′-AGAGTTTGATCMTGGCTCAG-3′ and 1492R 5′-GGTTACCTTGTTACGACTT-3′) were used for amplification. Genomic DNA was isolated using the Bacteria & Tissue DNA Isolation Kit (Eurx, Gdańsk, Poland). PCR was performed in a thermocycler ABI 9700 (Thermo Fisher Scientific, Warsaw, Poland) with thermostable polymerase OptiTaq (Eurx, Gdansk, Poland). The product was enzymatically purified with the ExoSAP kit (Thermo Fisher Scientific, Warsaw, Poland), and sequencing was performed using the BigDye Terminator v3.1 Cycle Sequencing Kit and the ABI 3730xl analyzer (Thermo Fisher Scientific, Warsaw, Poland), using primers 41F, 518R and 928F. The obtained consensus sequence was compared with the reference data in the NCBI 16S (Bacteria and Archaea) databases using the BLAST tool (http://blast.ncbi.nlm.nih.gov, Accessed on 10 February 2024) and the EzBioCloud platform (https://www.ezbiocloud.net/identify, Accessed on 10 February 2024).

### 3.2. Postbiotic Preparation

Before the preparation of postbiotics, strain B1, stored at −80 °C, was reactivated for 24 h at 37 °C under static conditions on MRS agar, according to previously described microaerophilic conditions (6% O_2_, 10% CO_2_, 84% N_2_). A single colony was transferred to 50 mL of MRS broth and incubated overnight at 37 °C, obtaining a late logarithmic phase culture. 4 mL of this culture (approx. 10^8^ CFU/mL) was then used to inoculate 200 mL of fresh MRS broth (2% *v*/*v*). After 24 h of incubation, the cell suspension was centrifuged (10,000 rpm, 10 min, room temperature), and the supernatant was separated and sterilized by vacuum filtration (Rapid Filtermax, PES membrane, 0.22 µm; TPP, Bionovo, Legnica, Poland). The filtered supernatant was used to prepare postbiotics in two variants: lyophilized and organic extracts. In the case of lyophilization, 1 L of the supernatant was cooled at −20 °C and then lyophilized (Alpha 2-4, Martin Christ Gefriertrocknungsanlagen GmbH, Osterode am Harz, Germany) for 72 h at 0.1 mbar and −60 °C. An average of 10 g of dry powder was obtained (*n = 3*), stored at −20 °C. Pure MRS broth was also lyophilized as a control. To obtain the organic extract, 1 L of the supernatant was extracted twice with ethyl acetate (1:1, *v*/*v*; PoCh, Gliwice, Poland). The organic phase was dehydrated with anhydrous sodium sulfate (Chempur, Piekary Śląskie, Poland), filtered (Whatman 1 filters, Merck, Poznań, Poland) and evaporated at 40 °C in a vacuum evaporator. An average of 2 g of an extract was obtained (*n = 3*), stored at −20 °C. In parallel, an extract was prepared from pure MRS broth as a control.

### 3.3. Identification of Organic Acids Using GC-MS

Postbiotics in the form of lyophilisates and organic extracts, obtained according to the procedures described earlier, as well as organic acid standards: hydroxyisocaproic acid, lactic acid, malonic acid, shikimic acid, succinic acid (Organic Acids Kit, Supelco, Merck, Poznań, Poland) were analyzed. Samples and standards were dissolved in ethanol at a concentration of 1 mg/mL (PoCh, Gliwice, Poland) and then evaporated to dryness at 40 °C in chromatography vials. Evaporated samples were derivatized by adding 100 µL of N,O-bis(trimethylsilyl)trifluoroacetamide (BSTFA; Sigma-Aldrich, Munich, Germany) and incubating at 50 °C for 45 min. After derivatization, the volume was made up to 1 mL with ethyl acetate. Qualitative analysis of extracts and lyophilized post-culture fluids of the LAB B1 strain was analyzed using a 7890A gas chromatograph equipped with a 5975C mass spectrometer (GC-MS, Agilent Technologies, Santa Clara, CA, USA). The injection volume was 1.6 µL of solution. The analysis was carried out in split mode (10:1); helium as a carrier gas flowed at a rate of 1.2 mL/min. The inlet temperature was 280 °C. The oven program included: 80 °C (2 min), increasing by 20 °C/min to 300 °C (3 min). The total analysis time was 16 min. The mass spectrometer was operated in full scan mode (range 25–450 amu). Identification of organic acids was performed based on retention times and mass spectral analysis and confirmed using the NIST 08 MS library. Quantitative analysis was based on an eight-point calibration curve that showed linearity in the range of 2 to 100 µg/mL (r = 0.997).

### 3.4. Antimicrobial Activity of Postbiotics

Antimicrobial activity of postbiotics was assessed using the broth microdilution method, according to the Clinical and Laboratory Standards Institute guidelines (CLSI, M07, 11th edition). The activity was tested against the following reference strains: *Staphylococcus aureus* ATCC 29213, *Escherichia coli* ATCC 25922, *Streptococcus pyogenes* ATCC 19615 and *Cutibacterium acnes* ATCC 6919. The analyses were performed in 96-well plates using MH broth (Becton Dickinson, Warsaw, Poland) for aerobic strains or BHI broth (Becton Dickinson, Warsaw, Poland) for *C. acnes*. Postbiotics (LLCFS and ELCFS) were diluted in the appropriate medium. The concentration range was 0.2–100 mg/mL (LLCFS) and 0.04–20 mg/mL (ELCFS). A bacterial suspension prepared in an appropriate medium was added to each well to obtain a final inoculum concentration of 5 × 10^5^ colony-forming units (CFU)/mL. The samples and appropriate biotic (medium inoculated with microorganism),abiotic controls (medium with postbiotics) and medium controls were incubated for 24 h at 37 °C (aerobic strains) or for 48 h under microaerophilic conditions (6% O_2_, 10% CO_2_, 84% N_2_) for the C. acnes strain. Absorbance was measured at 630 nm (Multiskan™ FC reader, Thermo Fisher Scientific). The MIC was defined as the lowest postbiotic concentration at which no growth was observed. To determine the MBC, 100 µL of suspension from wells without growth was plated onto MH agar or BHI agar (for *C. acnes*) and incubated for 24 h (aerobic) or 48 h (microaerophilic, *C. acnes*), respectively, at 37 °C. MBC was defined as the lowest postbiotic concentration leading to a complete inhibition of microorganism growth. MIC and MBC results were expressed in g/L.

### 3.5. Long-Time and Thermal Stability of Postbiotics

Thermal stability of postbiotics B1 was assessed based on the retention of antimicrobial activity after a 30 min exposure to the temperatures of 20 °C, 40 °C, 60 °C and 80 °C. The ELCFS (10 g/L) and LLCFS (50 g/L) were analyzed at concentrations corresponding to the values close to the MIC. The samples stored at refrigerated temperature (4 °C) were used as controls. After thermal treatment, residual activity against reference strains (*S. aureus*, *E. coli*, *S. pyogenes* and *C. acnes*), according to the procedure described in Section 3.4. Percentage residual activity was calculated using the formula:

$$RA\;\left(\%\right)=\frac{100-\%sample\;growth}{100-\%control\;growth}\;\times \;100\%$$

The results were expressed as a percentage of residual activity relative to the sample not exposed to elevated temperature.

Additionally, the antimicrobial stability of the postbiotics was assessed after 12 months of storage at −20 °C. For this purpose, MIC and MBC values for all tested strains were determined for both ELCFS and LLCFS samples stored under frozen conditions, by the procedure described in Section 3.4.

### 3.6. Biofilm Inhibition and Eradication

The ability of postbiotics obtained from strain B1 to inhibit biofilm was assessed against reference strains: *S. aureus*, *E. coli*, *S. pyogenes* and *C. acnes*. Two mechanisms of action were analyzed: (i) inhibition of biofilm formation and (ii) eradication of already formed biofilm. In the case of the inhibition of biofilm formation, postbiotics were used at concentrations equal to: MIC, 0.9×MIC, 0.8×MIC, 0.5×MIC and 0.1×MIC, determined earlier, as well as biotic, abiotic and medium controls. The study on microtiter plates was carried out according to the previously described methodology [71]. Clear, flat-bottomed, untreated 96-well polystyrene plates (Thermo Fisher Scientific, Warsaw, Poland) were prepared in the same manner as the procedure described in the section on the assessment of antimicrobial activity. After 24 h of incubation under appropriate conditions, the plates were stained with crystal violet according to the previously described methodology [71]. Staining allowed for the quantitative assessment of the level of biofilm formation in the presence of different concentrations of postbiotics. To assess the ability of postbiotics to eradicate preformed biofilm, concentration ranges of 0.04–20 mg/mL for the ELCFS and 0.2–100 mg/mL for the LLCFS were used. The procedure was performed according to the methodology described by Abruzzo et al. (2021) [72], with minor modifications. Biofilm was formed in 96-well microtiter plates by incubating bacterial suspension (200 µL, 5 × 10^5^CFU/mL) for 48 h under appropriate culture conditions. The biofilm was then treated with 200 µL of postbiotic B1 at the selected concentration and incubated for another 24 h. After incubation, similarly to the biofilm inhibition test, crystal violet staining was performed to quantify the biofilm residue.

### 3.7. Biofilm Visualization by Confocal Microscopy

The visualization of the effect of LAB B1 strain postbiotics on biofilm formation was performed in the Laboratory of Microscopic Imaging and Specialized Biological Techniques (Faculty of Biology and Environmental Protection, University of Lodz, Lodz, Poland) using a Leica TCS SP8 confocal laser microscope (Leica Microsystems, Wetzlar, Germany), equipped with plan-achromatic objectives with 63× (water immersion) and 100× (oil immersion) magnification. Four reference strains were used for the study: *S. aureus*, *E. coli*, *S. pyogenes* and *C. acnes*, cultivated according to the previously described procedure on 24-well glass-bottom microtiter plates (NEST^®^, GenoPlast Biotech, Rokocin, Poland), adapted for confocal microscopy. Cells were exposed to selected concentrations of postbiotics that had previously been shown to significantly inhibit biofilm formation: 2.5 mg/mL for the ELCFS and 25 mg/mL for the LLCFS. Biotic controls were run in parallel, including cultures not exposed to postbiotics. Biofilm cell viability was assessed using the LIVE/DEAD™ BacLight™ Bacterial Viability Kit (Thermo Fisher Scientific, Warsaw, Poland), according to the manufacturer’s instructions. After incubation with postbiotics and in control samples, the contents of the wells were washed three times with phosphate-buffered saline (PBS, BioShop, Burlington, VT, Canada) to remove planktonic cells. Then, 200 µL of PBS containing 1 µL of Syto 9 dye and 0.6 µL of propidium iodide were added to each well. After 30 min incubation in the dark at 37 °C, the dyes were removed, and the wells were washed three times with PBS. To enable imaging, the wells were covered with a thin layer of PBS (approx. 2 mm). Fluorescence was recorded at excitation/emission wavelengths: 480/500 nm for Syto 9 and 490/635 nm for propidium iodide.

### 3.8. Cytotoxicity

The effect of postbiotics on mammalian cells was assessed using mouse fibroblast line L929 (ATCC CCL-1), according to the international standard ISO 10993-5:2009 [53]. Cells (2 × 10^4^ cells/well) were cultured in 96-well microtiter plates (Nunclon Delta Surface, Rochester, NY, USA) in Roswell Park Memorial Institute medium (RPMI-1640), supplemented with 10% bovine serum (FBS; HyClone Cytiva, Malboroug, MA, USA) and a mixture of penicillin (100 U/mL) and streptomycin (100 µg/mL) (Sigma Aldrich, Darmstadt, Germany). Incubation was carried out in 5% CO_2_ at 37 °C for 24 h. After this time, the culture medium was removed and 100 µL of fresh medium containing postbiotics was added. ELCFS and LLCFS were first dissolved in sterile water and then diluted in the medium to final concentrations of 0.02–10 mg/mL for the ELCFS and 0.1–50 mg/mL for the LLCFS, corresponding to the concentrations previously shown to inhibit the growth and biofilm formation of the tested microorganisms. Culture medium without the addition of postbiotics was used as a negative control, while the positive control was medium with the addition of 10 µL of DMSO (BioShop, Burlington, VT, Canada). The cells were incubated for another 24 h under the same conditions. After the incubation was completed, the medium was removed and 100 µL of fresh medium and 20 µL of 3-(4,5-dimethylthiazol-2-yl)-2,5-diphenyltetrazolium bromide (MTT, 5 mg/mL; Merck, Poznań, Poland) solution were added to each well. The plates were incubated for 4 h at 37 °C. The medium was then removed and 200 µL of DMSO was added to dissolve the formed formazan crystals. Absorbance was measured at 595 nm using a Multiskan™ FC microplate reader spectrophotometer (Thermo Fisher Scientific, Pudong, Shanghai, China). Based on the obtained results, the percentage of cell viability was calculated relative to the negative control (Cell lines without active substance added).

### 3.9. Statistical Analysis

All experiments were performed in four independent replicates (*n* = 4). Results are presented as means ± ±SD. Normality of data distribution was assessed using the Shapiro–Wilk test. Statistical significance of differences between groups was assessed using the nonparametric Mann–Whitney U test. Analyses were performed using GraphPad Prism v. 10.4.1 (GraphPad Software, San Diego, CA, USA).

## 4. Conclusions

In this study, we demonstrated that postbiotics obtained from the *Lactiplantibacillus pentosus* B1 strain isolated from beetroot sourdough exhibited a significant antimicrobial and antibiofilm activity against bacteria associated with skin infections. The two forms of preparations—ELCFS and LLCFS—differed in their range of activity and cytotoxicity profile, with ELCFS showing a stronger bactericidal effect, while LLCFS was characterized by a higher cellular safety. Importantly, in both cases concentrations causing partial inhibition and eradication of biofilm did not show cytotoxicity towards fibroblasts. High thermal stability and long-term durability indicate the potential of using both forms of postbiotics as antimicrobial substances. Chemical analyses using the GC-MS method revealed the presence of organic acids, which are most probably responsible for the observed biological effects. The diverse composition and biological activity of postbiotics depending on the method of their preparation emphasizes the importance of optimizing the production process.

For the first time, a comparison of the biological activity of two postbiotic preparations of *L. pentosus* B1 postbiotics obtained from a natural fermented plant source has been presented. The obtained results constitute the basis for further preclinical studies aimed at using these compounds as safe alternatives to classical antibiotics in the treatment and prevention of skin infections.

Despite the promising results, this study has certain limitations. First, all experiments were conducted in vitro, limiting the ability to directly relate the results to in vivo conditions. Second, the chemical analysis was focused on identifying organic acids using GC–MS, which does not allow for a full characterization of other potentially active metabolites, such as bacteriocins, biosurfactants or antimicrobial peptides. Third, the antimicrobial and antibiofilm activity of individual identified metabolites was not assessed, making it impossible to determine their individual contribution to the observed biological effect. Filling these gaps in future studies, especially in in vivo models, will allow for a more comprehensive assessment of the application potential of the obtained postbiotics in cosmetic formulations.

## Figures and Tables

**Table 1:** MIC and MBC of fresh and 12-month-stored ELCFS and LLCFS against selected bacterial strains (g/L).

Strain	MIC [g/L]				MBC [g/L]			
	ELCFS		LLCFS		ELCFS		LLCFS	
	Fresh	Stored	Fresh	Stored	Fresh	Stored	Fresh	Stored
*S. aureus* ATCC 29213	10	10	50	50	20	20	100	100
*E. coli* ATCC 25922	10	10	25	25	10	10	50	50
*S. pyogenes* ATCC 19615	10	10	50	50	20	20	-	-
*C. acnes* ATCC 6919	10	10	50	50	20	20	100	100

**Table 2:** Thermal stability of postbiotics ELCFS and LLCFS assessed by %RA against selected bacterial strains after a 30 min incubation at 20, 40, 60, 80 °C compared to a control stored at 4 °C. The results are presented as mean ± SD (*n* = 4). Statistically significant differences compared to control were denoted as * (*p* < 0.05).

ELCFS [10 g/L]				
Strain	20 °C	40 °C	60 °C	80 °C
*S. aureus* ATCC 29213	99.98 ± 0.02%	99.96 ± 0.15%	99.75 ± 0.33%	99.84 ± 0.08%
*E. coli* ATCC 25922	99.98 ± 0.01%	100.04 ± 0.01%	99.67 ± 0.12%	99.78 ± 0.00%
*S. pyogenes* ATCC 19615	99.99 ± 0.07%	99.45 ± 0.03%	99.61 ± 0.22%	99.55 ± 0.05%
*C. acnes* ATCC 6919	100.04 ± 0.12%	29.66 ± 19.13% *	30.77 ± 11.90% *	50.29 ± 2.88% *
LLCFS [50 g/L]				
Strain	20 °C	40 °C	60 °C	80 °C
*S. aureus* ATCC 29213	100.31 ± 0.07%	100.02 ± 0.08%	99.03 ± 0.06% *	99.44 ± 0.22% *
*E. coli* ATCC 25922	99.98 ± 0.28%	100.05 ± 0.26%	100.11 ± 0.08%	100.05 ± 0.08%
*S. pyogenes* ATCC 19615	99.68 ± 0.58%	100.43 ± 0.15%	100.10 ± 0.27%	100.07 ± 0.00%
*C. acnes* ATCC 6919	99.55 ± 0.91%	97.81 ± 0.15% *	95.84 ± 0.66% *	96.40 ± 0.31% *

**Table 3:** Concentrations of organic acids in postbiotics. All data are presented as mean ± standard deviation (SD) (*n* = 4).

	[mg/L]	
Organic Acid	ELCFS	LLCFS
Hydroxyisocaproic acid	11.63 ± 2.47	1.33 ± 0.07
Lactic acid	350.64 ± 53.35	98.53 ± 30.21
Malonic acid	12.63 ± 2.13	3.10 ± 0.02
Shikimic acid	1.56 ± 0.01	1.59 ± 0.04
Succinic acid	4.45 ± 1.10	0.66 ± 0.07

## Data Availability

The original contributions presented in this study are included in the article. Further inquiries can be directed to the corresponding authors.
